# Association of select vitamin D receptor gene polymorphisms with the risk of tobacco-related cancers – a meta-analysis

**DOI:** 10.1038/s41598-019-52519-5

**Published:** 2019-11-05

**Authors:** Lukasz Laczmanski, Izabela Laczmanska, Felicja Lwow

**Affiliations:** 1 Hirszfeld Institute of Immunology and Experimental Therapy, Polish Academy of Science, Weigla 12, Wroclaw, Poland; 2 Genetics Department, Wroclaw Medical University, Marcinkowskiego 1, Wroclaw, Poland; 3 Team of Health Promotion, Faculty of Physiotherapy, University School of Physical Education, Paderewskiego 35, Wroclaw, Poland

**Keywords:** Cancer prevention, Tumour biomarkers, Risk factors

## Abstract

The observed increase in morbidity and mortality due to tobacco-related cancers, especially those in the respiratory system and esophagus, is becoming a public health challenge. Smoking cigarettes is one of the main risk factors predisposing individuals to many types of cancers. The aim of this study was to determine the role of select vitamin D receptor (VDR) gene polymorphisms as risk factors in tobacco-related cancers. The MEDLINE and ResearchGate databases were used to search for articles up to June 2017, and 12 articles including 26 studies concerning FokI, ApaI, TaqI and BsmI polymorphisms and lung, neck, head, esophageal and oral cancers were chosen. In total, 5 113 cases and 5 657 controls were included in the pooled analysis. We found a significant relationship between tobacco-related cancers and the occurrence of the “t” allele in the TaqI polymorphism of VDR. The occurrence of the “t” allele reduced the risk of tobacco-related cancers by 17% (OR = 0.83, 0.72–0.96 95% CI, p-value = 0.0114). Our analysis revealed that there is a correlation between the TaqI polymorphism of VDR and the risk of tobacco-related cancers.

## Introduction

Smoking cigarettes, a low level of physical activity and a poor diet are the main lifestyle risk factors for diseases that are typical in developed and developing countries^1–3^. The association between increased risk of chronic diseases (including cancers) and cigarette smoking is well known and thoroughly described^4–6^. Tobacco-associated cancers are a broad group that include cancers of the oral cavity, throat, larynx, esophagus, trachea, pancreas, bladder, kidney, stomach, liver, colon, and cervix as well as myeloid leukemia^5^. Although the effects of cigarette smoke components on the development of cancer such as breast cancer and prostate cancer have been confirmed, the main group of tobacco-related cancers are respiratory system cancers such as those occurring in the larynx, bronchial tubes, lung and oral cavity. Lung cancer is the leading cause of cancer-related mortality, especially in the male population, and smoking habits are the primary risk factor for lung cancer^7–10^. However, lung cancer does not develop in all smokers and can occur in nonsmokers. Furthermore, because cancers are multifactorial diseases, it has been suggested that other factors such as exposure to other carcinogens (e.g., asbestos), poor diet, past lung diseases (e.g., tuberculosis, chronic bronchitis) and genetic factors may be significant in the development of lung cancer^11–13^.

The combustion products of cigarettes contain many toxic chemical components, such as formaldehyde, lead, arsenic, benzene, carbon monoxide, and nitrosamines, that have been previously described as playing roles in DNA damage. DNA repair pathways, such as nucleotide excision repair and base excision repair (NER/BER), nonhomologous end joining (NHEJ) and homologous recombination (HR), were reported to be strongly influenced by smoking^14^. Some gene variants involved in these pathways and other cellular tasks can predispose to or even cause many cancers. The role of gene mutations and polymorphisms are still studied because of their importance for the etiology, development and progression of diverse cancers^15^. Additionally, the active form of vitamin D has been shown to exhibit apoptosis induction, anti-proliferative and anti-inflammatory effects, invasion and metastasis inhibition of cancer cells^16–20^.

Vitamin D carries out its functions by interacting with the vitamin D receptor (VDR). The *VDR* gene is located on the q arm of chromosome 12 and consists of a promoter, six regulatory sequences and seven exons that code for six protein domains. Only some single nucleotide polymorphisms (SNPs) can regulate VDR expression or activity, among many SNPs of the VDR gene^16^. In this paper, the following *VDR* SNPs were chosen: *FokI* (rs10735810), located in the coding region, which results in a polymorphic protein form shorter by first three, amino acids; *BsmI* (rs1544410); *ApaI* (rs7975232); and *TaqI* (rs731236). These SNPs are located in the 3′-untranslated region and are responsible for mRNA stability, which may influence *VDR* expression or activity (http://www.ncbi.nlm.nih.gov/snp). Low vitamin D status is associated with cancers^17–20^. In turn it is known that vitamin D has anti-inflammatory effects and improves body defense mechanisms, while cigarette smoking is a pro-inflammatory factor and weakens body defense mechanisms^21^. Therefore, vitamin D status may play an important role in tobacco-related cancers. *VDR* gene polymorphisms have been suggested to be correlated with the risk of different cancers due to their role in the modulation of the antiproliferative effect of vitamin D^22^.

The aim of this study was to determine whether select *VDR* polymorphisms may be risk factors in tobacco-related cancers.

## Results

Twelve articles including 26 studies were found that described the connection between *VDR* polymorphisms and four different cancers (oral, head and neck, esophagus, and lung) and met the inclusion criteria. In total, 5 113 cases and 5 657 controls were included in the pooled analysis (Supplementary Table S1). We chose five papers regarding the *FokI* polymorphism^13,23–27^, six regarding the *BsmI* polymorphism^23,25,28–31^, six regarding the *ApaI* polymorphism^23,25,28–30,32^ and nine regarding the *TaqI* polymorphism^23–29,33–35^. The Newcastle-Ottawa Scale (NOS) score ranged between 5 and 8 (Supplementary Table S1). It means that every article is of high or moderate quality.

The results of the Egger and the Begg and Mazumdar asymmetry tests for the FokI, BsmI, ApaI and TaqI polymorphisms were nonsignificant (Table 1). Funnel plots are presented in the Supplementary Materials. The time-lag bias was analyzed by cumulative analysis (Supplementary Material M1). We can conclude that there are no statistically significant differences in OR value when comparing individual years of publications.

**Table 1:** Results of the Egger and Begg-Mazumdar asymmetry tests.

	Egger b0 [95% CI]	Egger p-value	Begg Mazumdar Z	Begg Mazumdar p-value
*FokI*	−0.5823 [−5.3135–4.1489]	0.7215	−0.9767	0.3298
*BsmI*	−2.1990 [−5.3114–0.9434]	0.1240	−0.9798	0.3272
*TaqI*	−0.6131 [−2.1276–0.9573]	0.3703	−0.9651	0.3345
*ApaI*	−0.0631 [−5.7755–5.6494]	0.9770	−0.1879	0.8510

CI – confidence interval; a p-value < 0.05 was considered statistically significant.

We applied analysis of LD between 4 selected SNPs. We used data gathered in the 1000 Genomes Project specific for Caucasian and Asian populations (these were predominant in our study). As can be seen in Supplementary Materials M1 Fig. 5 and Table 1, 3 out of 4 SNPs are very strongly associated, so the number of effective independent markers (and thus the independent comparisons) was set to 2. Consequently, the significance threshold was set to p-value ≤ 0.05/2 = 0.025.

### *FokI* polymorphism

**Figure 1:**
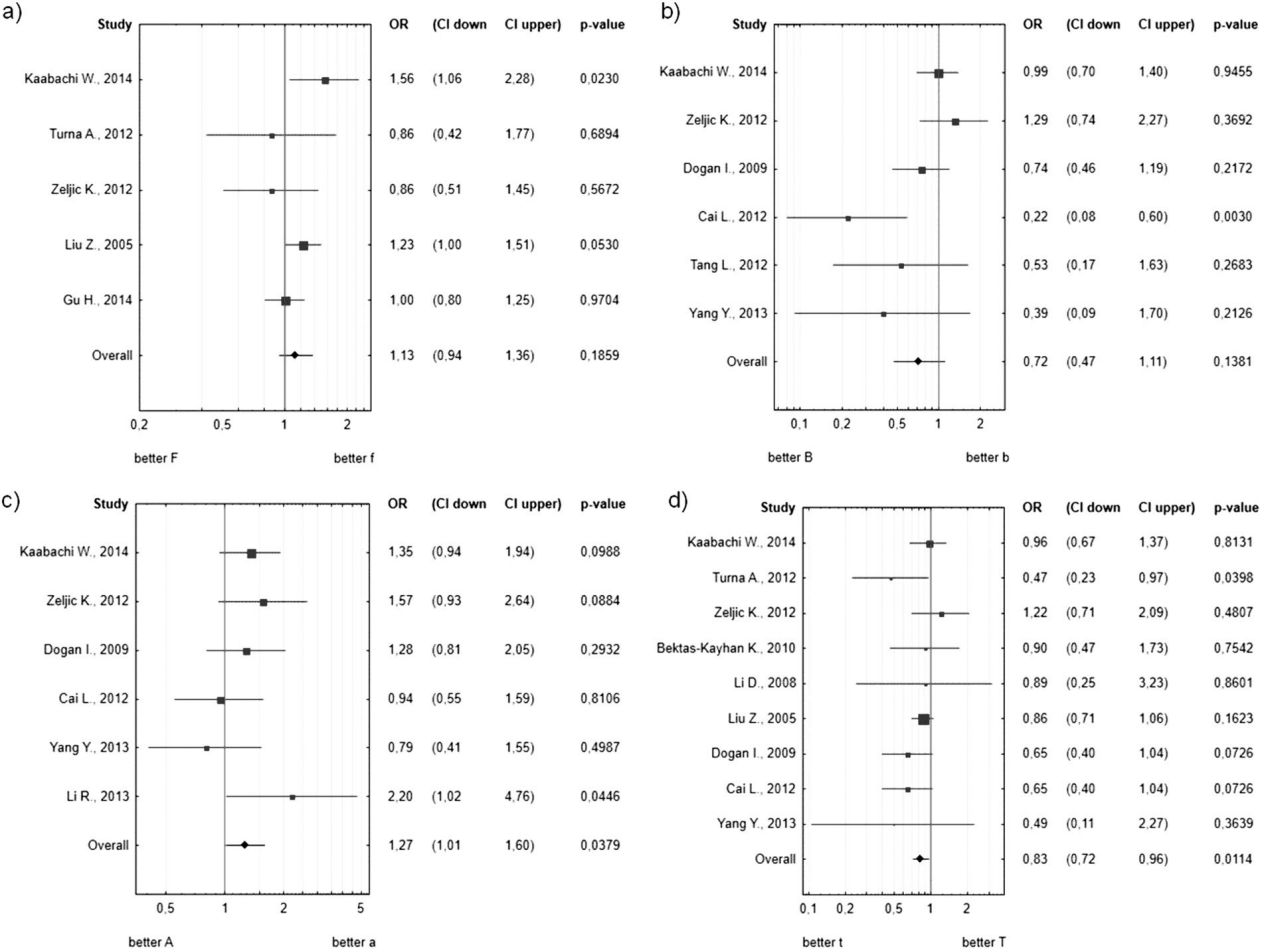
Forest plots estimating the effect of VDR polymorphisms on the risk of tobacco-related cancers. (**a**) *FokI* polymorphism, (**b**) *BsmI* polymorphism, (**c**) *ApaI* polymorphism, (**d**) *TaqI* polymorphism. The estimate of the OR (odds ratio of the polymorphic form with respect to wild type – WT) and its 95% CI are plotted with a box and a horizontal line, respectively. Filled boxes: pooled OR and its 95% CI.

The cumulative OR for the “F” allele of the *FokI VDR* polymorphism in the tobacco-related cancer group was 1.13 (0.94–1.36 95% CI) and was not statistically significant (p-value = 0.1859) (see Fig. 1a). Heterogeneity between studies was not significant (Q = 5.967, I2 = 32.97%, p = 0.2016). The I^2^ below 40% suggested that heterogeneity is medium; therefore all the articles can be analyzed in our meta-analyses.

**Figure 2:**
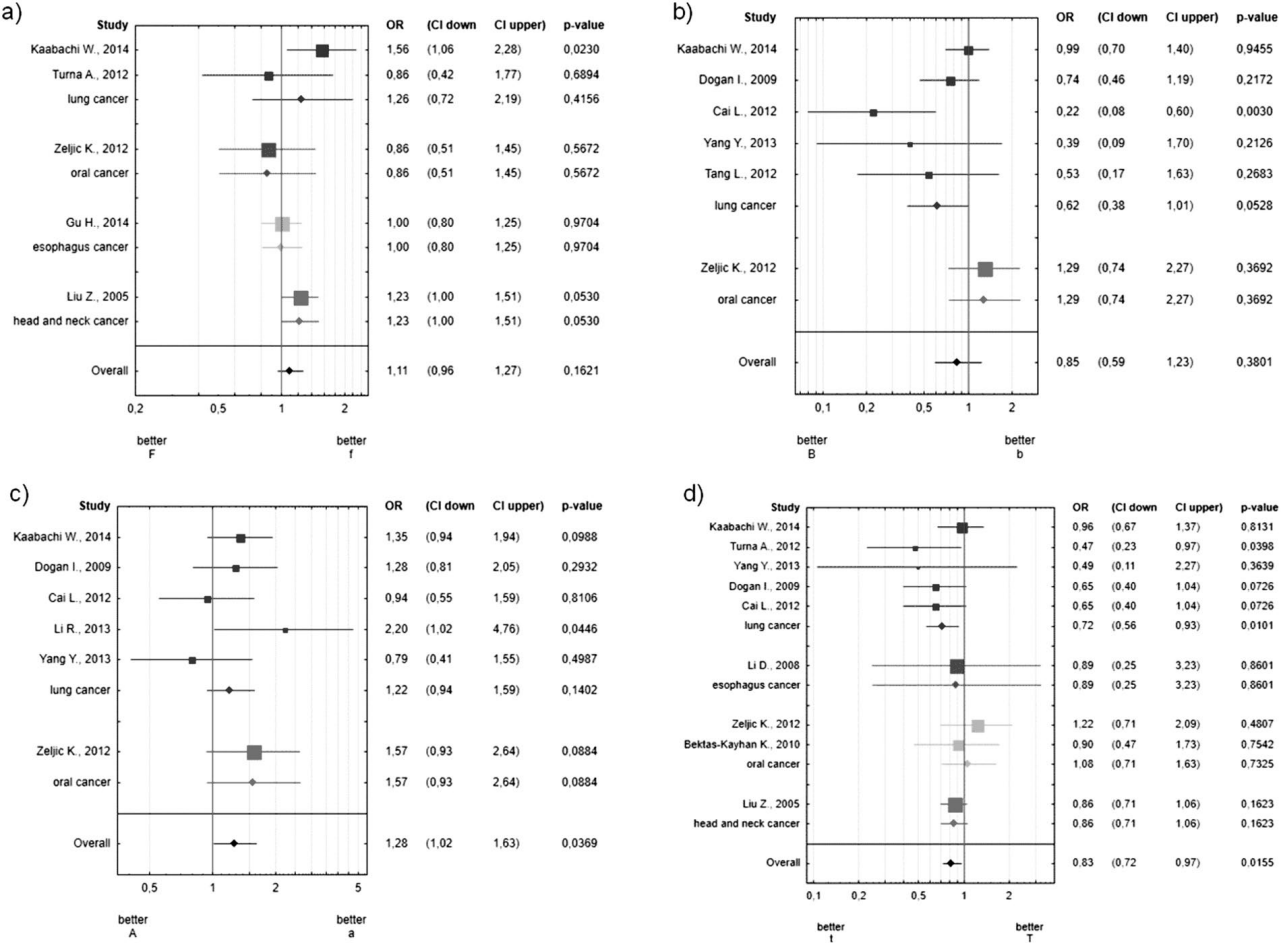
Forest plots estimating the cumulative effect of VDR polymorphisms on the risk of tobacco-related cancers stratified by cancer site. (**a**) *FokI* polymorphism, (**b**) *BsmI* polymorphism, (**c**) *ApaI* polymorphism, (**d**) *TaqI* polymorphism. The estimate of the OR (odds ratio of polymorphic form with respect to wild type – WT) and its 95% CI are plotted with a box and a horizontal line, respectively. Filled boxes: pooled OR and its 95% CI.

All the publications were grouped according to the localization of the cancer (lung, esophagus, oral, head and neck cancers). According to our criterion that p < 0.025 is statistically significant, we did not observe statistically significant OR for all cancer locations (p-value = 0.0530) (see Fig. 2a).

### *BsmI* polymorphism

The cumulative OR for the “B” allele of the *BsmI* polymorphism in the tobacco-related cancer group was 0.72 (0.47–1.11 95% CI) and was not statistically significant (p-value = 0.1381) (see Fig. 1b). After classifying the publications according to the site of the cancer (lung, esophagus, oral, head and neck cancers), we observed a tendency but not a significant relationship between the *BsmI* polymorphism and lung cancer. The “B” allele may decrease the risk of lung cancer by 38% (OR = 0.62 0.38–1.01 95% CI, p-value = 0.0528) (see Fig. 2b Heterogeneity between studies was significant (Q = 11.904, I2 = 58%, p = 0.0361). Based on the funnel plot (Supplementary Fig. 2) we designated the Cai *et al*. 2012 article as the outlier publication. The heterogeneity was not significant after rejecting outlier publication (Q = 4.565, I2 = 12%, p = 0.3349), but it is important that the summary OR did not change significantly and was similar (OR = 0.90 0.69–1.19 95%CI p-value = 0.4643).

### *TaqI* polymorphism

The cumulative OR for the “t” allele of the *TaqI VDR* polymorphism in the tobacco-related cancer group was 0.83 (0.72–0.96 95% CI) and was statistically significant (p-value = 0.0114) (see Fig. 1d). The data from the nine publications show that the “t” allele reduces the tobacco-related cancer risk by 17%. Next, we grouped the data according to the site of the cancer (lung, esophagus, oral, head and neck cancers). We observed that the “t” allele reduced the risk of lung cancer by 28% (OR = 0.72, 0.56–0.93 95% CI, p-value = 0.0101), and we did not observe any statistically significant effect on the risk of developing other cancers (see Fig. 2d).

Heterogeneity between studies was not significant (Q = 7.722, I2 = 0.0001%, p = 0.4611). The I^2^ below 1% suggested that heterogeneity is very small, therefore all the articles can be analyzed in our paper.

### *ApaI* polymorphism

The cumulative OR for the “A” allele of the *ApaI VDR* polymorphism in the tobacco-related cancer group was 1.27 (1.01–1.60 95% CI). According to our criterion that p < 0.025 is statistically significant we didn’t observe a significant relationship between presence of allele “A” of the *ApaI* polymorphism and risk of tobacco-related cancers (p-value = 0.0379) (see Fig. 1c). Heterogeneity between studies was not significant (Q = 5.892, I2 = 15.14%, p = 0.3169). The I^2^ below 40% suggested that heterogeneity is medium; therefore all the studies can be analyzed.

After grouping publications according to the site of the cancer, we did not observe any statistical significance between the *ApaI* polymorphism and cancer location (lung, esophagus, oral, head and neck cancers) (p-value = 0.0369) for all site of cancers (see Fig. 2c).

## Discussion

Genetic alterations in important cell biochemical pathways may play roles in cancer development together with environmental or lifestyle factors^15^. Likewise, tobacco-related cancers are strongly dependent on environmental factors and habits such as tobacco smoking, and air pollution are also influenced by some genetic factors^13,36^.

Recently, one of the most intensively studied genes in connection to the risk of cancers has been that of the vitamin D receptor. Vitamin D has been reported to regulate the expression of over 150 metabolic genes, and vitamin D deficiency may be correlated with a higher risk of developing cancers, diabetes, and autoimmune or cardiovascular diseases^16^. In our study, we focused on the effect of four VDR polymorphisms and we assessed the impact on the risk of developing cancers of the respiratory system and esophagus. We analyzed publications between 2005 and 2014, but most of them were published between 2010 and 2014. The time-lag bias analysis did not show any significant differences for all polymorphisms in a particular year. All analyzed publications were written in English.

Through the meta-analysis of 26 polymorphism studies, we revealed statistically significant ORs only for *TaqI* polymorphism but not for *ApaI*, *BsmI* and *FokI*. The cumulative OR for the “t” allele of the *TaqI VDR* polymorphism in the tobacco-related cancer group was 0.83 (0.72–0.96 95% CI), which was statistically significant (p-value = 0.0114). The data from nine publications (focused on lung cancer, esophagus cancer, oral, head and neck cancer) showed that the occurrence of the “t” allele reduced the risk of tobacco-related cancers by 17%. This observation was similar to those previously reported in other publications concerning breast and prostate cancers^37,38^.

After sorting publications according to the site of the cancer, we observed that the “t” allele reduced the risk of lung cancer by 28% (OR = 0.72, 0.56–0.93 95% CI, p-value = 0.0101) but did not have any statistically significant effects on cancers at other sites (i.e., esophagus, oral, head and neck). In previous meta-analyses by other authors, the association between the *TaqI* (24 439 cases and 26 406 controls) and *ApaI* (12 542 cases and 13 574 controls) genotypes and the risk of cancer was investigated with different cancer sites, i.e., 17 articles on prostate cancer, 11 articles on breast cancer, 8 articles on colorectal cancer, 6 articles on skin cancer, 3 articles on ovarian cancer and 17 articles on other cancers^39^. *TaqI* and *ApaI v*ariant genotypes were not associated with the risk of cancer in general. A significant effect was observed only for colorectal cancer; the risk of developing colorectal cancer in patients with the tt *TaqI* genotype was 43% higher than for those with other genotypes^39^.

Our findings were consistent with the conclusion drawn in another study that specific *VDR* polymorphisms are associated with the risk of non-small-cell lung cancer (NSCLC), which supports the hypothesis that there is a connection between the vitamin D pathway and the risk of NSCLC in a population of patients with specific haplotypes^40^. Although the authors did not confirm that higher levels of endogenous vitamin D were associated with a lower risk of NSCLC in the Han Chinese population, they concluded that cigarette smoking may lead to vitamin D deficiency. Rai *et al*.^38^ described a connection between *TaqI* and *BsmI* polymorphisms and the risk of lung cancer (NSCLC)^38^. Our results confirm the relation between the *TaqI* polymorphism and a lower risk of developing respiratory cancers observed in “t” allele carriers. In “B” allele carriers in the *BsmI* genotype and “A” allele in the ApaI genotype, we observed a tendency, but the results did not reach statistical significance.

It is known that “BAt” haplotype of the VDR polymorphisms is more expressed then “baT” haplotype and it confers a better response to the ligand (the active form of vitamin D)^41^. On the other hand, smoking or certain air pollution factors may reduce the serum vitamin D level and reduce its efficiency. Therefore a more highly expressed VDR gene may reduce the negative effect of a lower level of vitamin D.

### Limitations

Our work has some limitations. In our meta-analysis, we did not compare smokers and nonsmokers, although it is known that compared with nonsmokers, smokers have a 20-fold increased risk of developing certain cancers, e.g., lung cancer, but we did not find any information about it in analyzed papers. Information about smoking is important for cancer prevention and health promotion, including screening for cancer, modifying lifestyles, and improving vitamin D status.

We found that the “t” allele of the *TaqI VDR* polymorphism increased the risk of developing tobacco-related cancer (lung) by approximately 28%, but we did not analyze the number of years of addiction to smoking or the number of years from potential smoking cessation to onset of the disease. Seven years of smoking abstinence reduces lung cancer mortality by an amount comparable to that produced by screening by computed tomography^40^. Furthermore, our meta-analysis was based on a few studies, some of which were published between 2005 and 2017. We did not focus on the genetics associated with ancestries either, because of the small numbers of articles, but on the other hand we evaluated homogeneous Caucasians and inhabitants of Asia.

Another limitation is the small number of analyzed publications. We analyzed data from only 26 studies, but it should be emphasized that these studies involve over 5 000 patients and controls. This means that the problem will require further research and analysis.

The conclusion from the main results is that our paper focuses on very important aspects of publication bias and genetic risk factors associated with tobacco related cancer. This is the first such publication in our opinion.

## Conclusion

The *TaqI* polymorphism of *VDR* is associated with the risk of tobacco-related respiratory system and esophagus cancers. OR reduced by 17% is observed in “t” allele carriers.

## Materials and Methods

A systematic literature search was planned to conduct a meta-analysis of observational studies. The MEDLINE and ResearchGate databases were used to search for articles up to June 2017 using the following terms: “*VDR* (All Fields) AND (“polymorphism, genetic” (MeSH Terms) OR (“polymorphism” (All Fields) AND “genetic” (All Fields)) OR “genetic polymorphism” (All Fields) OR “polymorphism” (All Fields)) AND (“neoplasms” (MeSH Terms) OR “neoplasms” (All Fields) OR “cancer” (All Fields))”. Reference lists and conference reports were also reviewed. In total, 408 publications were found, from which the studies that compared cancer patients with a healthy control group were chosen. Meta-analyses were excluded. 12 articles but 26 polymorphism studies concerning *FokI*, *ApaI*, *TaqI* and *BsmI* polymorphisms and lung, neck and head, esophagus, and oral cancers were chosen. Odds ratios with 95% confidence intervals were used to assess the strength of associations between the polymorphisms of VDR and the cancer risk in the described populations. The search strategy is reported according to the PRISMA (http://www.prisma-statement.org/) reporting guidelines (see flow diagram in Supplementary Diag. S1). Quality rating was assessed by the Newcastle-Ottawa Scale (NOS)^42^. It was performed by two independent researchers. A study rating of 7–9 means high quality, 5–6 means medium quality, and below 4 stars means low quality.

The number of alleles was calculated according to the formula NA = n*q, where n is the total number of genotypes in the group, and q is the probability of the allele (“F” and f for *FokI*, “B” and “b” for *BsmI*, “A” and “a” for *ApaI* and “T” and “t” for *TaqI*)^23,37^.

The fixed-effects model and the DerSimonian-Laird random-effects model (with weights based on the inverse variance) were used to calculate summary odds ratios (ORs), and both within- and between-study variations were considered. Publication bias was analyzed using funnel plots of asymmetry, the Begg and Mazumdar test and the Egger test. Heterogeneity between studies was evaluated using the Cochran Q test and I^2^ estimates. Cumulative analysis for all polymorphisms by year of publication was prepared.

To estimate the significance level we first counted the effective number of independent markers (see Lee *et al*.^43^ for details). In brief, we measured linkage disequilibrium (LD) for 4 selected SNPs (data gathered from the 1000 Genomes Project, https://www.ncbi.nlm.nih.gov/pubmed/?term=26139635). Three out of 4 SNPs were very strongly associated (LD > 0.97); therefore the number of effective independent markers (and thus the independent comparisons) was set to 2. Consequently, the significance threshold was set to a p-value ≤ 0.05/2 = 0.025. All statistical analyses were performed using Statistica ver. 10 software (StatSoft, USA) with the Medical Package.

### Statement of significance

In our opinion this is the first meta-analysis summarized correlation between VDR polymorphisms and the risk of tobacco-related cancers.

## Supplementary information

Supplementary Materials M1

Supplementary Table S1

Supplementary Diag S1

PRISMA 2009 checklist
