# Supplementary material for: Association of select vitamin D receptor gene polymorphisms with the risk of tobacco-related cancers – a meta-analysis
Source: Sci Rep. 2019 Nov 5;9:16026. doi: 10.1038/s41598-019-52519-5 (PMC6831593; doi:10.1038/s41598-019-52519-5)

**Association of select vitamin D receptor gene polymorphisms with the risk of tobacco-related cancers – a meta-analysis**

Lukasz Laczmanski^1)*^, Izabela Laczmanska^2)^ and Felicja Lwow^3)*^

1. Hirszfeld Institute of Immunology and Experimental Therapy, Polish Academy of Science, Weigla 12, Wroclaw, Poland
2. Genetics Department, Wroclaw Medical University, Marcinkowskiego 1, Wroclaw, Poland
3. Team of Health Promotion, Faculty of Physiotherapy, University School of Physical Education, Paderewskiego 35, Wroclaw, Poland

***Corresponding authors**: Lukasz Laczmanski, professor PAS, Hirszfeld Institute of Immunology and Experimental Therapy, Polish Academy of Science, Weigla 12, 53-114 Wroclaw, Poland; e-mail: [lukasz.laczmanski@iitd.pan.wroc.pl](mailto:lukasz.laczmanski@iitd.pan.wroc.pl), phone: +48 605 314 478; professor Felicja Lwow, Team of Health Promotion, Faculty of Physiotherapy, University School of Physical Education, Paderewskiego 35, Wroclaw, Poland, email: [felicitas1@wp.pl](../../../../C:%5CUsers%5CŁukasz%5CDocuments%5Cartykuły%5Cmetaanaliza%5CVDR_vs_tobacco_cancer%5CSR%5Crevision%5Cfelicitas1@wp.pl)

**Supplementary Figure 1.** VDR polymorphism *FokI* in tobacco related cancer. Funnel Plot. OR – odds ratio and 95% CI.

**Supplementary Figure 2.** VDR polymorphism *BsmI* in tobacco related cancer. Funnel Plot. OR – odds ratio and 95% CI.

**Supplementary Figure 3.** VDR polymorphism *TaqI* in tobacco related cancer. Funnel Plot. OR – odds ratio and 95% CI.

**Supplementary Figure 4.** VDR polymorphism *ApaI* in tobacco related cancer. Funnel Plot. OR – odds ratio and 95% CI.

**Supplementary Figure 5.** Pairwise analysis of LD between 4 SNPs


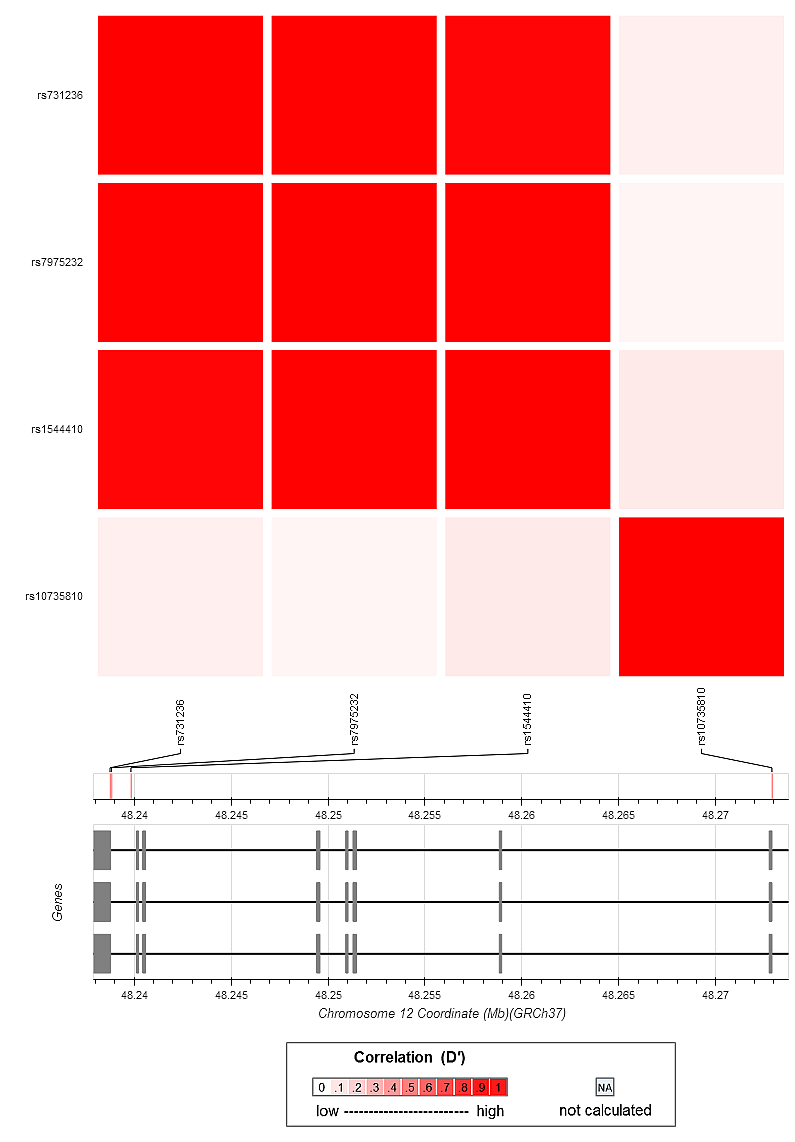


**Supplementary Table 1.** LD matrix for selected SNPs

|  | **rs731236** | **rs7975232** | **rs1544410** | **rs10735810** |
| --- | --- | --- | --- | --- |
| **rs731236** | **1.0** | 0.998 | 0.979 | 0.063 |
| **rs7975232** | 0.998 | **1.0** | 0.998 | 0.041 |
| **rs1544410** | 0.979 | 0.998 | **1.0** | 0.083 |
| **rs10735810** | 0.063 | 0.041 | 0.083 | **1.0** |

**Supplementary Figure 6.** Cumulative analysis for polymorphisms: A-FokI, B-BsmI, C-ApaI and D-TaqI . OR – odds ratio and 95% CI.


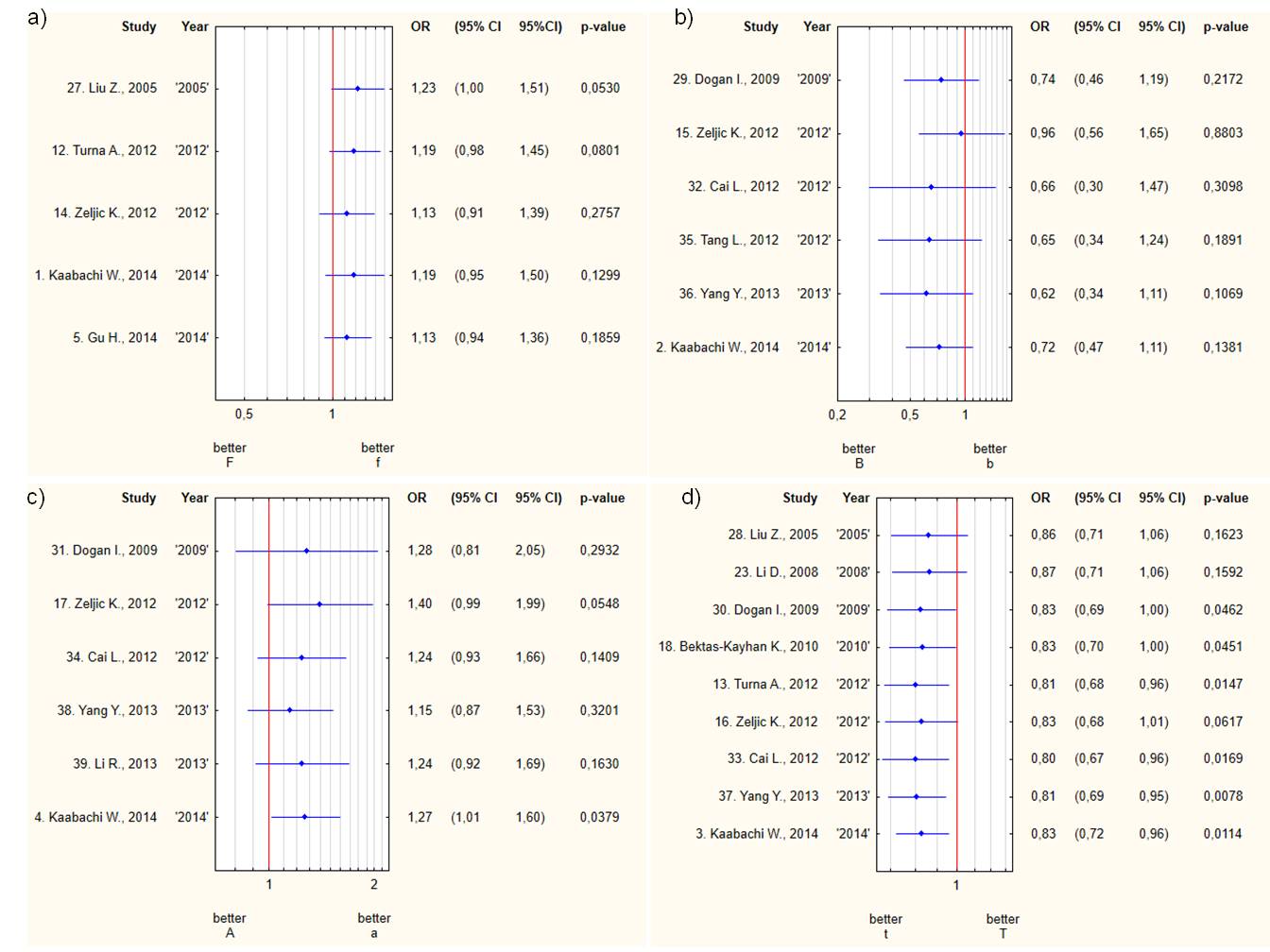

Supplement: Supplementary file 1 — Supplementary Materials M1 [file 41598_2019_52519_MOESM1_ESM.doc]
