# Supplementary material for: Association of select vitamin D receptor gene polymorphisms with the risk of tobacco-related cancers – a meta-analysis
Source: Sci Rep. 2019 Nov 5;9:16026. doi: 10.1038/s41598-019-52519-5 (PMC6831593; doi:10.1038/s41598-019-52519-5)
Supplement: Supplementary file 3 — Supplementary Diag S1 [file 41598_2019_52519_MOESM3_ESM.doc]

**Association of select vitamin D receptor gene polymorphisms with the risk of tobacco-related cancers – a meta-analysis**

Lukasz Laczmanski^1)*^, Izabela Laczmanska^2)^ and Felicja Lwow^3)*^

1. Hirszfeld Institute of Immunology and Experimental Therapy, Polish Academy of Science, Weigla 12, Wroclaw, Poland
2. Genetics Department, Wroclaw Medical University, Marcinkowskiego 1, Wroclaw, Poland
3. Team of Health Promotion, Faculty of Physiotherapy, University School of Physical Education, Paderewskiego 35, Wroclaw, Poland

*Corresponding authors: Lukasz Laczmanski, professor PAS, Hirszfeld Institute of Immunology and Experimental Therapy, Polish Academy of Science, Weigla 12, 53-114 Wroclaw, Poland; e-mail: [lukasz.laczmanski@iitd.pan.wroc.pl](mailto:lukasz.laczmanski@iitd.pan.wroc.pl), phone: +48 605 314 478; professor Felicja Lwow, Team of Health Promotion, Faculty of Physiotherapy, University School of Physical Education, Paderewskiego 35, Wroclaw, Poland, email: [felicitas1@wp.pl](../../../../C:%5CUsers%5CŁukasz%5CDocuments%5Cartykuły%5Cmetaanaliza%5CVDR_vs_tobacco_cancer%5CSR%5Crevision%5Cfelicitas1@wp.pl)


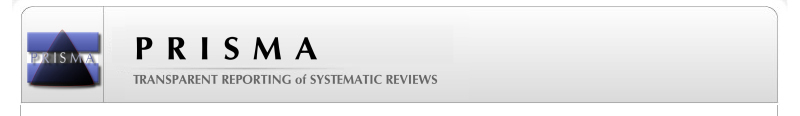
**PRISMA 2009 Flow Diagram**

**Screening**

**Included**

**Eligibility**

**Identification**

Records identified through database searching
(n = 408 )

Additional records identified through other sources
(n =0 )

Records after duplicates removed
(n = 408 )

Records screened
(n = 406 )

Records excluded

(other meta-analizys excluded)
(n = 2 )

Full-text articles assessed for eligibility:

Articles concerning *FokI, ApaI, TaqI* and *BsmI* polymorphisms and lung, neck and head, esophagus, and oral cancers were chosen.
(n =26 )

Full-text articles excluded due to the lack of complete data
(n = 380 )

Studies included in qualitative synthesis
(n = 26 )

Studies included in quantitative synthesis (meta-analysis)
(n = 26 )
